# ROLE OF INCISIONAL VACUUM THERAPY IN ENDOPROSTHETIC BONE RECONSTRUCTION SURGERY

**DOI:** 10.1590/1413-785220233104e260330

**Published:** 2023-07-31

**Authors:** ANDRÉ MATHIAS BAPTISTA, ANDRÉ FERRARI DE FRANÇA DE CAMARGO, MARCELO TADEU CAIERO, JOSÉ SAINT CLAIR DE SOUSA TORRES, JORGE HENRIQUE NARCISO, MARCELA ROCHA DIAS SILVA

**Affiliations:** 1 Universidade de Sao Paulo, Faculdade de Medicina, Hospital das Clinicas, Instituto de Ortopedia e Traumatologia IOT HCFMUSP, Grupo de Oncologia Ortopedica, Sao Paulo, SP, Brazil.

**Keywords:** Negative Pressure Wound Therapy, Prostheses and Implants, Osteosarcoma, Tratamento de Ferimentos com Pressão Negativa, Próteses e Implantes, Osteossarcoma

## Abstract

Reconstructive surgery with endoprostheses is the chosen method for treating bone malignancies. Postoperative infections are frequent complications, and their treatment involves prolonged hospital stays and antibiotic therapy. Among the advancements aimed at reducing the rate of postoperative infection, the use of incisional negative pressure therapy (iNPT) has shown promising results, with no reports in the literature regarding its use in patients with such conditions.

**Objective:**

To evaluate the effectiveness of iNPT in reducing postoperative complications in surgeries for resection of bone tumors associated with modular endoprosthesis reconstruction.

**Methods:**

Retrospective case series of 16 patients diagnosed with osteosarcoma, who underwent resection and reconstruction with endoprosthesis associated with iNPT during the postoperative period. Follow-up was performed for a period of six months, and the evaluated outcomes were the incidence of postoperative infection and complications of the surgical wound.

**Results:**

The use of iNPT for a postoperative period of seven days resulted in only three (18.7%) cases of postoperative infection. No cases of wound dehiscence, seroma formation, or hematoma at the surgical site were observed.

**Conclusion:**

The rate of surgical wound complications in our case series is lower than that reported in most of the literature, and iNPT appears to be an efficient way to reduce the rate of local complications in reconstructive surgeries with endoprosthesis after resection of bone malignancies. ***Level of Evidence III, Retrospective Study.***

## RESUMO

A cirurgia reconstrutiva com endopróteses é o método escolhido no tratamento de malignidades ósseas. As infecções pós-operatórias são complicações frequentes, e seu tratamento envolve internações e antibioticoterapia prolongadas. Entre os avanços que visam reduzir a taxa de infecção pós-operatória, o uso da terapia com pressão negativa incisional (TPNi) vem mostrando resultados promissores, não havendo relatos na literatura de seu emprego em pacientes com tal quadro.

**Objetivo:**

Avaliar a eficácia da TPNi em reduzir complicações pós-operatórias em cirurgias de ressecção de tumores ósseos associadas à reconstrução com endopróteses modulares.

**Métodos:**

Série de casos retrospectiva de 16 pacientes diagnosticados com osteossarcoma, submetidos à ressecção e reconstrução com endoprótese associada à TPNi durante o pós-operatório. Foi realizado seguimento por um período de seis meses e os desfechos avaliados foram incidência de infecção pós-operatória e complicações da ferida operatória.

**Resultados:**

O uso da TPNi por um período pós-operatório de sete dias resultou em apenas três (18,7%) casos de infecção pós-operatória. Não foram observados casos em que ocorreu deiscência da ferida operatória, formação de seromas ou hematomas no sítio cirúrgico.

**Conclusão:**

A taxa de complicações de ferida operatória em nossa série de casos é menor que a da maior parte da literatura, e a TPNi parece ser uma forma eficiente de reduzir a taxa de complicações locais em cirurgias reconstrutivas com endoprótese após ressecção de malignidades ósseas. **Nível de Evidência III, Estudo Retrospectivo.**

## INTRODUCTION

Osteosarcomas are rare primary malignant neoplasms of the bone tissue. Currently, the main form of treatment for these tumors consists of resection of the lesion and reconstructive surgery using endoprostheses.^1^ The primary advantages of this method include limb preservation, rapid function restoration with early rehabilitation, good long-term functional outcomes, and wide availability in specialized services for the treatment of musculoskeletal neoplasms. ^(^ ^2^ Disadvantages include material wear, which leads to aseptic loosening, fractures, and periprosthetic infections. ^(^ ^3^

Due to the increased overall survival rate among patients with orthopedic tumors, implant failure has become one of the primary complications in treating bone sarcomas, with implant-related infection being the most frequent. ^(^ ^4^ Surgical site infections are associated with significant morbidity and cost during their follow-up. ^(^ ^5^

Considering the impact on the patient’s quality of life and the financial burden on the healthcare system, ^(^ ^6^ ^),(^ ^7^ the reduction of postoperative infections has been the focus of numerous studies. As a result of advancements in material quality, reduced surgical time, improved surgical techniques, periodic glove changes, and other enhancements, postoperative infection rates associated with the use of endoprostheses have decreased. ^(^ ^4^

However, some studies still report periprosthetic infection rates of 15 to 20% in the early years of postoperative period. ^(^ ^8^ It is known that persistent incisional drainage occurs in 1 to 3% of patients undergoing arthroplasty surgeries, resulting in an increased infection risk of 29 to 42% for each day the condition persists. In this context, there is a significant focus on optimizing care for the surgical wound and the use of negative pressure therapy (NPT). ^(^ ^9^

The application of NPT originated centuries ago in traditional Chinese medicine, and its use in Western traditional medicine was approved by the Food and Drug Administration only in 1995 for the treatment of wounds deemed incurable. Today, its application has been extended to include the management of chronic wounds, acute wounds, subacute wounds, traumatic wounds, burns, dehiscence, coverage failures, diabetic foot ulcers, pressure ulcers, among others. ^(^ ^10^ ^),(^ ^11^

One of the modalities of interest and worth delving into for this study is incisional negative pressure therapy (iNPT), which is used in surgical wounds undergoing primary closure. This therapy is applied directly to the incision site using polyurethane or polyvinyl alcohol foam, a gas-permeable adhesive tape, a “TRAC pad,” a connecting tube, and a vacuum device that maintains a continuous negative pressure of 125 mmHg. ^(^ ^11^ The benefits of iNPT include acting as a barrier to the external environment and protecting the incision from contaminants, reducing tension forces on the surgical wound, minimizing stress on the suture line, optimizing tissue perfusion, and reducing the formation of hematomas and seromas. ^(^ ^9^ The effect on bacterial bioburden has shown conflicting findings in the literature, with more recent studies demonstrating an increase in bioburden without affecting wound healing. ^(^ ^12^ The main drawback of the method is its high cost.

Although widely studied, there are few studies on iNPT in the field of orthopedic oncology. This study aims to describe the treatment outcomes of patients undergoing oncologic resection and reconstruction with knee and hip endoprostheses, along with the use of iNPT, at the Orthopedics and Traumatology Institute of the Hospital das Clínicas, Faculty of Medicine, University of São Paulo (IOT - HCFMUSP).

## METHODS

This study is a retrospective case series aimed at reporting the results obtained by the Department of Orthopedic Oncology at IOT-HCFMUSP using Incisional Negative Pressure Therapy following oncologic resection surgery and reconstruction with endoprostheses in patients treated from January 2018 to December 2020 at the quaternary healthcare center. This study has been approved by the hospital’s Ethics and Research Committee under protocol number 1.529/22.279.

The study included patients who had reached skeletal maturity, were literate, diagnosed with osteosarcomas, underwent resection and reconstruction with endoprostheses, and received postoperative iNPT. The exclusion criteria were as follows: clinical and radiographic follow-up of less than six months, use of iNPT for less than five days, insufficient data in medical records, and refusal to sign an informed consent form.

All surgeries were performed by the authors (CMT, BAM, and/or CAFF), and the data - including age, gender, tumor type and location, surgical treatment specifics, duration of iNPT use, length of hospital stay, surgical wound complications, overall postoperative complications, as well as subsequent necessary treatments - were collected from the electronic medical record system and available imaging exams of the participating patients in the study.

The primary analyzed outcome was the occurrence of postoperative infection, which was determined based on the presence of inflammatory changes with or without secretion, along with laboratory alterations such as increased inflammatory markers and/or positive culture from deep surgical site material. As secondary outcomes, other local complications of the surgical wound such as dehiscence and fluid collections were evaluated.

The results will be presented descriptively using distribution measures such as mean, standard deviation, and percentage, calculated using the PASW Statistics 18.0 software (SPSS Inc.). Chicago, USA) in a number of cases.

## RESULTS

Incisional negative pressure therapy (iNPT) was used in a total of 16 patients over the course of these two years, including 5 women and 11 men, with a mean age of 44 years. All patients underwent iNPT for a total of seven days (Table 1). Only two patients had diseases other than neoplastic.

**Table 1:** Mean age, gender, length of hospital stay, time of incisional negative pressure therapy.

Number of patients	Age	Length of hospital stay*	Time of iNPT*	Gender
***n***	***Mean (SD)***	***Mean (SD)***	***Mean (SD)***	***n (%)***
16	44.1 (16.8)	12.1 (6.5)	19.3 (14.5)	Women = 5 (31.2)
				Men = 11 (68.8)

*Measured in days.

n: number; SD: standard deviation; iNPT: incisional negative pressure therapy.

Among the performed reconstructions, there were two (12.5%) hip endoprostheses, five (31.2%) total femur endoprostheses, eight (50%) knee endoprostheses, and one (6.2%) proximal tibia endoprosthesis. Out of these, ten were primary reconstructive procedures, and six were revision surgeries. After a 6-month outpatient follow-up, only three (18.7%) patients presented postoperative infection, with no occurrence of other surgical wound complications such as dehiscence, and hematoma or seroma formation. In most patients - 10 (62.5%) - at least one surgical procedure had already been performed in the location of the osteosarcoma. The above information is presented in Table 2, among the patients who presented or not with postoperative infection.

**Table 2:** Presence of comorbidities, previous infections, and previous surgeries.

	Comorbidities	Previous infections	Previous surgeries on tumor topography	Revision of the primary endoprosthesis
	***n (%)***	***n (%)***	***n (%)***	***n (%)***
**No postoperative infection (n = 13)**	1 (7.6)	1 (7.6)	8 (61.5)	4 (30.7)
**Postoperative infection (n = 3)**	1 (33.3)	2 (66.6)	2 (66.6)	2 (66.6)

n: number.

## DISCUSSÃO

In our case series, we observed a predominance of men, similar to the studies conducted by Theil et al., ^(^ ^3^ but with a significantly higher mean age of 44, which is considerably higher compared to the aforementioned study with mean age of 21.

In the systematic review conducted by Thornley et al., ^(^ ^13^ osteosarcoma was identified as the most frequent primary malignancy among patients, excluding cases of metastasis. The study also reported a high rate of surgical re-intervention following tumor resection and primary reconstructive surgery. In these cases, it was observed that only 5% of the reoperations occurred due to tumor recurrence, whereas the remaining 95% were due to postoperative local complications. Mechanical causes such as periprosthetic fracture, implant failure, and aseptic loosening were more frequent, followed by infectious causes. ^(^ ^13^

In general, we found an infection rate of 18.7%, slightly lower than the 22% presented by Theil et al. ^(^ ^3^ for cases of primary approach. When comparing these rates to revision surgeries, we observed a value of 37%, which is slightly lower than the 39% reported in the aforementioned study. Regarding non-infectious complications of surgical wounds, no cases of dehiscence or other complications were found, contrasting with an approximate incidence of 17% reported in the previous study. ^(^ ^3^

To our knowledge, no studies have compared the outcomes of using iNPT in reconstructive surgeries following tumor resection. Studies on primary arthroplasties have shown that iNPT can reduce the risk of infection by up to four times. Similar findings have also been reported in patients with orthopedic trauma, which are also high-risk cases for surgical wound complications. In these cases, the use of iNPT resulted in a reduction of more than five times in the risk of infection, from 28% to 5.4%.^9^

A meta-analysis conducted by Hyldig et al., ^(^ ^14^ which included various studies on orthopedic surgeries in trauma and reconstruction, supports the findings that the use of iNPT reduces the risk of surgical site infection, dehiscence, seroma formation, and other complications. However, the number needed to treat (NNT) reached up to 25, considering that the cost associated with it is more than 10 times that of a simple dressing, which would not justify the routine use of this therapy. ^(^ ^14^ It is worth noting that several studies included in this meta-analysis presented methodological issues and a short follow-up period, limiting the analysis of the quality of evidence and extrapolations regarding cost-effectiveness.

The study conducted by Cooper et al. ^(^ ^15^ defends the routine use of iNPT in high-risk patients for postoperative wound complications since the rates of endoprosthesis preservation in cases of deep surgical site infection are low, and the cost of reoperation and continuing care in these patients is high. The cost-effectiveness analysis conducted by Nherera et al. ^(^ ^16^ demonstrated cost savings of $10,293.00 to $11,296.00 per treated patient, resulting from the savings in the treatment of local complications and their repercussions.

## CONCLUSION

In this case series, we observed a lower rate of surgical site infection than expected when compared with the findings in the literature for reconstructive surgeries with endoprosthesis after resection of malignant bone tumors, as well as the absence of other complications such as dehiscence and fluid collections. Despite the high cost of incisional negative pressure therapy, the use of this therapeutic strategy in high-risk wounds seems to be justified. Considering the low sample size of this study, further prospective and randomized studies are necessary to corroborate with our hypotheses. However, our data indicate that iNPT can reduce the risks of infection and complications associated with bone resection and reconstruction with endoprosthesis.
